# Vascular change and opposing effects of the angiotensin type 2 receptor in a mouse model of vascular cognitive impairment

**DOI:** 10.1038/jcbfm.2014.221

**Published:** 2014-12-10

**Authors:** Martina Füchtemeier, Marie P Brinckmann, Marco Foddis, Alexander Kunz, Chrystelle Po, Caterina Curato, Ulrich Dirnagl, Tracy D Farr

**Affiliations:** 1 Department of Experimental Neurology, Center for Stroke Research Berlin (CSB), Charité Universitätsmedizin Berlin, Berlin, Germany; 2 German Center for Neurodegenerative Diseases (DZNE), Berlin, Germany; 3 Imagerie par Résonance Magnétique Médicale et Multi-Modalités, Université Paris Sud, Orsay, France; 4 German Rheumatism Research Center (DRFZ), A Leibniz Institute, Berlin, Germany; 5 Excellence Cluster NeuroCure, Berlin, Germany; 6 German Center for Cardiovasular Diseases (DZHK), Berlin, Germany

**Keywords:** angiotensin II receptor type 2, diffusion tensor imaging, mouse hypoperfusion, perfusion weighted imaging, vascular cognitive impairment

## Abstract

Our aims were to assess the spatiotemporal development of brain pathology in a mouse model of chronic hypoperfusion using magnetic resonance imaging (MRI), and to test whether the renin-angiotensin system (RAS) can offer therapeutic benefit. For the first time, different patterns of cerebral blood flow alterations were observed in hypoperfused mice that ranged from an immediate and dramatic to a delayed decrease in cerebral perfusion. Diffusion tensor imaging revealed increases in several quantitative parameters in different brain regions that are indicative of white-matter degeneration; this began around 3 weeks after induction of hypoperfusion. While this model may be more variable than previously reported, neuroimaging tools represent a promising way to identify surrogate markers of pathology. Vascular remodelling was observed in hypoperfused mice, particularly in the anterior part of the Circle of Willis. While the angiotensin II receptor type 2 agonist, Compound 21 (C21), did not influence this response, it did promote expansion of the basilar artery in microcoil animals. Furthermore, C21-treated animals exhibited increased brain lymphocyte infiltration, and importantly, C21 had opposing effects on spatial reference memory in hypoperfused and sham mice. These results suggest that the RAS may have a role in vascular cognitive impairment.

## Introduction

Vascular cognitive impairment (VCI) refers to a group of cognitive disorders that are presumed to share a vascular origin; together they represent the second leading cause of dementia after Alzheimer's disease. The exact pathophysiology of VCI is not understood, but the incidence and known risk factors (hypertension, atherosclerosis, advanced age, and obesity) are on the rise. Despite identification of VCI as a top research priority for the next 10 years by the Stroke Progress Review Group at the National Institute of Neurological Disorders and Stroke, the development of appropriate preclinical animal models is lagging.^1^ It is generally accepted that there is a causal link between chronic brain hypoperfusion and cognitive impairment in a rat model of bilateral common carotid artery occlusion,^2^ and a comparable mouse model was developed in which both carotid arteries were made stenotic using small microcoils.^3^ The authors reported white-matter lesions in the corpus callosum at 14 days after microcoil implantation, which is a useful hallmark of certain subtypes of VCI. Since then, a few reports have described similar findings, as well as spatial working memory impairments after 1 to 2 months of hypoperfusion.^4, 5^ Another group reported hippocampal degeneration when survival times were extended to 8 months,^6^ and, by bilaterally varying the degree of stenosis, one group observed microinfarcts within the most severely deprived cortex.^7^

Nevertheless, a great deal of work is still required to characterize this model, identify pathophysiologic mechanisms that are relevant to certain aspects of the human condition, and to find therapeutic strategies. Therefore, the purpose of the present study was twofold. First, we aimed to establish a timeline of neurodegeneration using noninvasive, longitudinal neuroimaging, thus providing surrogate measures of neuropathology that would be equally applicable to humans. Second, we aimed to counteract the deleterious effects of brain hypoperfusion by pharmacologically modulating the renin-angiotensin system (RAS). Angiotensin II is a hormone that induces vasoconstriction and increases blood pressure via stimulation of the angiotensin II type 1 receptor. Angiotensin II type 1 receptors are widely expressed and have a detrimental role in cardiovascular and neurologic disease. Angiotensin II type 2 receptors (AT2Rs) are expressed in lower levels in regions of the brain that are involved in cognition, where they oppose the actions of angiotensin II type 1 receptors and are generally thought to provide the protective arm of the RAS.^8^ The specific nonpeptide, orally active AT2R agonist Compound 21 (C21) has already been shown to reduce cognitive decline in a mouse model of Alzheimer's disease^9^ and has been suggested to promote regeneration and reduce inflammation,^10^ thus, it represents an excellent candidate for use in VCI.

## Materials and methods

### Animals and Experimental Design

All experiments were performed in accordance with the German Animal Welfare Act and approved by the Landesamt für Gesundtheit und Soziales. Male C57/BL6 mice (10 weeks of age) were housed in a temperature (22±2°C), humidity (55±10%), and light (12/12 hours light/dark cycle) controlled environment. Animals were given *ad libitum* access to food and water throughout the duration of the experiments and monitored daily. No differences were observed in terms of the general condition, appearance, and behavior of the mice after surgery. All mice were active, grooming, feeding, and drinking post operatively and virtually no weight loss was exhibited.

**Figure 1:**
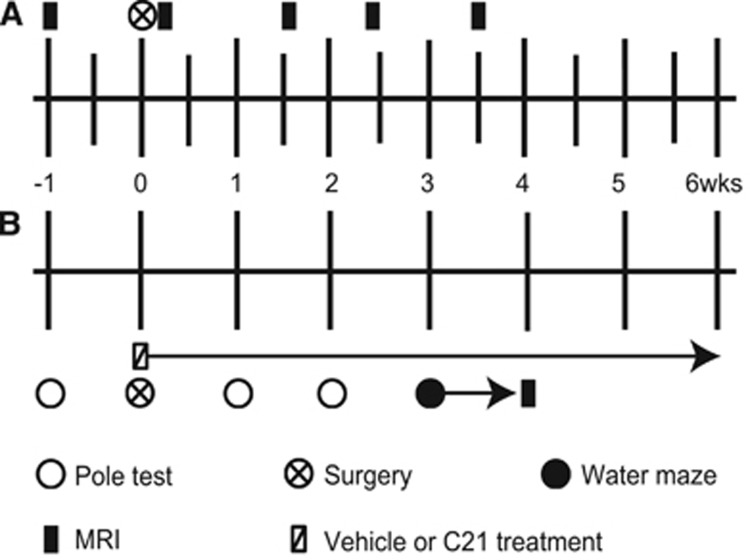
Timelines of the experimental design. (**A**) First experiment: T_2_-weighted imaging and cerebral blood flow (CBF) were measured before and at 4 and 24 hours, and 11, 17, and 25 days after microcoil implantation. Diffusion tensor imaging (DTI) was performed at the final three timepoints. (**B**) Second experiment: Mice were tested for sensorimotor function in the pole test and 1 week later underwent microcoil implantation or sham surgery. At this time, they began receiving daily intraperitoneal injections of Compound 21 (C21) (0.3 mg/kg) or vehicle. They were tested in the pole test and a place task in the Morris water maze was used to assess spatial learning and reference memory. Finally, mice underwent anatomic magnetic resonance imaging (MRI).

During the first experiment (Figure 1A) eight mice that underwent microcoil implantation were imaged repetitively (before and at 4 and 24 hours, and 11, 17, and 25 days after microcoil implantation) for quantitative estimation of cerebral blood flow (CBF). Diffusion tensor imaging (DTI) was also acquired at the final three timepoints. Exclusion criteria stipulated that any animal with an adverse outcome that met local criteria for euthanasia would be excluded as well as any magnetic resonance imaging (MRI) measurement in which severe motion artefact was present. No measurements or animals were excluded from the analysis.

During the second experiment (Figure 1B), which was performed in two separate rounds, mice were divided into four different groups based on housing arrangements: (1) Sham, Vehicle (*n*=22), (2) Sham, C21 (*n*=11), (3) Microcoil, Vehicle (*n*=26), and (4) Microcoil, C21 (*n*=13). The experimenters were blinded to the condition of the animals. Mice were trained in the pole test and tested 1 week before, and at 7 and 14 days after microcoil implantation, or the corresponding sham procedure. Also starting at the time of surgery, mice began receiving daily intraperitoneal injections of 0.3 mg/kg of C21,^9^ or vehicle (Aqua ad iniectabilia). At 21 days, the animals were trained in a standard hidden platform (place) task in the Morris water maze, with a probe trial on the final day. Exclusion criteria for the water maze stipulated that any mice that did not learn the protocol were excluded from the analysis (one animal each from groups 1 and 3). Additionally, a technical malfunction on one of the probe trial days resulted in the exclusion of the animals scheduled for testing that day (seven mice from group 1 and four from group 3). Anatomic MRI experiments were also performed at the final timepoint on a subset of mice from each group: Sham, Vehicle (*n*=14), Sham, C21 (*n*=3), Microcoil, Vehicle (*n*=20), and Microcoil, C21 (*n*=11). Exclusion criteria for the imaging results stipulated that any mice considered outliers (more than 1.5X the interquartile range) would be excluded from the analysis (one animal from group 1). At the conclusion of the experiments, tissue was harvested for flow-cytometry analysis.

### Chronic Hypoperfusion Via Bilateral Common Carotid Artery Stenosis

Anesthesia was achieved using isoflurane in a 70:30 nitrous oxide:oxygen mixture and core body temperature was maintained at 37.0±0.2°C using an automated heat blanket with temperature feedback. Animals were placed in a supine position, a midline incision was made in the neck, and the sternomastoid muscles were carefully retracted on both sides to expose the carotid arteries. Chronic hypoperfusion was induced by isolating the carotid arteries and wrapping microcoils (180 *μ*m inner diameter, Sawane Spring Company, Hamamatsu, Japan) around them. The corresponding sham procedure was performed up to the point of microcoil wrapping. All muscles and glands were guided back into place, the incision sutured, and local anesthetic applied to the wound before recovery.

### Behavioral Testing

Sensorimotor function was assessed with the pole test, as previously described.^11^ Briefly, preoperative training was performed for 3 days with four trials per day. Animals were tested before and at 7 and 14 days after surgery. For each trial, mice were placed facing upward on the top of a 60-cm pole and the time required for the animals to both turn to face downward and descend to the home cage were recorded. Data from the four daily trials were pooled. A standard place task in the Morris water maze was used to assess spatial learning and reference memory. A circular maze (120 cm diameter) was filled with opaque water to cover a submerged escape platform (fixed location). Three daily trials were performed for each animal over 7 consecutive days. A trial consisted of lowering the animal into the pool from different starting points and allowing them 90 seconds to find the platform, or be collected. Trials were spaced 10 minutes apart and the animals were allowed to rest under a heat lamp in between trials. Escape latency (seconds), total distance travelled (cm), and swim speed (cm/s) were measured by a computerized tracking system (VideoMot, TSE Systems, Bad Homburg, Germany). Data from the three daily trials were pooled. At 8 days, a probe trial was performed in which the platform was removed and the mice were allowed to swim for 90 seconds. The total time spent in the former target quadrant (s) was assessed.

### Magnetic Resonance Imaging Measurements

All MRI experiments were conducted on a 7T system (Bruker BioSpin, Ettlingen, Germany). Anesthesia was again achieved using isoflurane (as above) in spontaneously breathing mice, and body temperature and respiration rate were monitored with MRI compatible equipment (Small Animal Instruments, Inc., Stony Brook, NY, USA).

During the first experiment, radio frequency transmission was achieved with a 72-mm diameter quadrature resonator actively decoupled to a mouse quadrature surface coil (Bruker BioSpin, Ettlingen, Germany). A RARE (rapid acquisition relaxation enhancement) *T*_2_ sequence (repetition time (TR)/echo time (TE): 5,000/10 ms, 8 echoes, RARE factor: 2, resolution: 156 *μ*m^2^, 16 minutes) was used to measure *T*_2_. The RARE sequence was always acquired first to allow the animal's body temperature and respiration rate to stabilize after induction of anesthesia. Subsequently, no adjustments were made to the anesthetic levels during the CBF measurement, as isoflurane is known to influence CBF. A single slice (1 mm) FAIR (flow-sensitive alternating inversion recovery) echo planar imaging (EPI) sequence (TR/TE: 10,000/10 ms, 10 inversion time (TIs) (30 to 2,500 ms), resolution: 195 *μ*m^2^, 13 minutes) was used to measure CBF together with a 2 mm 180° hyperbolic secant (sech80) inversion pulse (15 ms). Degeneration of the white matter was examined using an EPI DTI sequence (TR/TE: 4,000/29 ms, 4 segments, 6 *b* values (0 to 2,000 s/mm^2^) with 5 *b*0 images, 30 gradient directions, gradient duration (*δ*) 7 ms, gradient separation (Δ) 15 ms, resolution: 156 *μ*m^2^ isotropic, 40 minutes).

During the second experiment, a 20-mm diameter quadrature volume coil was used for radio frequency transmission and reception (RAPID Biomedical, Rimpar, Germany); a volume coil is necessary for homogenous signal during angiography measurements. The imaging protocol consisted of a *T*_2_-weighted multislice multiecho sequence (TR/TE: 3,000/10.5 ms, 16 echoes, resolution: 98 *μ*m^2^, 9 minutes), and a 3D time of light angiography sequence (TR/TE: 15/2.5 ms, *α*: 20&ring;, resolution: 98 *μ*m^3^, 6 minutes).

### Image Analysis

During the first experiment, *T*_2_ and the different quantitative DTI parameter maps were calculated using the Paravision software (Bruker BioSpin, Ettlingen, Germany) and exported to ImageJ freeware (National Institutes of Health). Several volumes of interest (VOIs) were chosen: the entire cortex, striatum, corpus callosum, and internal capsule. Each of these structures was manually delineated on a slice-by-slice basis on the *T*_2_-weighted images; this created several individual regions of interest (ROIs) for each structure. Subsequently, the ROIs were transferred to the FA maps and distortions in the EPI images were accounted for by manually adjusting the individual ROIs wherever necessary. Finally, integrated density was used to extract the different quantitative parameters across each VOI, thus providing one quantitative parameter value per structure. A custom-written ImageJ Plugin was used to fit *T*_1_ in the selective and nonselective FAIR experiments, and subsequently calculate CBF. In the case of the single slice CBF map, resolution only permitted identification of two ROIs: the striatum and the cortex.

During the second experiment, quantitative *T*_2_ maps were calculated again using the Paravision software and data were rescaled by a factor of 10 and exported into FSL software (Analysis Group, FMRIB, Oxford, UK). For the *T*_2_ images, the BET (brain extraction tool) was used to segment the brain, and the process was completed manually. The FLIRT (FMRIB's linear image registration tool) was used to co-register the segmented images to a mouse brain template (average of 10 C57/BL6 mouse brains). Subsequently, the FAST (FMRIB's automated segmentation tool) was used to segment the ventricles. All resulting images were exported into ImageJ. A binary mask was created for VOIs that corresponded to the striatum and the corpus callosum. The mask was used to extract *T*_2_ across both VOIs in each animal. Overall ventricle size and brain volumes were measured, and the determinant from the coregistration process was used to extrapolate the original volume. Angiography images were also exported to FSL and the FLIRT was used to coregister them to the first acquired data set. Subsequently, the images were exported back into ImageJ and the same volume was selected to build a maximum intensity projection of the Circle of Willis using a custom-written Plugin. A threshold that corresponded to roughly half of the signal in all the images (14,000) was chosen to exclude most of the brain tissue and convert the maximum intensity projection to a binary image. The images were visually inspected and voxels that did not correspond to the vasculature were manually reset to 0. The overall size of the Circle of Willis and basilar artery was calculated from the binary image (by counting voxels with a value of 1), and the volumes were corrected using the determinant.

### Preparation of Mononuclear Cells from Brain and Blood for Flow-Cytometry Analysis

Mice were euthanized at the conclusion of the experiments with intraperitoneal thiopental (25 mg) and blood samples were extracted from the heart before perfusion with physiologic saline. Blood was incubated with saline lysis buffer for 3 minutes at room temperature to eliminate erythrocytes. Brains were removed and digested with collagenase (Worthington Biochemical, Lakewood, NJ, USA) followed by mechanical disaggregation. Mononuclear cells were isolated on a Percoll gradient (GE Healthcare, Freiburg, Germany) by centrifugation for 20 minutes at 450 *g* at room temperature. Cells between 40% and 100% Percoll phases were collected, washed with phosphate-buffered saline, and centrifuged.

After blocking of Fc receptors (2.4G2), antibodies against CD3 (145-2C11), CD4 (GK1.5), CD8a (53-6.7), CD19 (1D3), CD11b (M1/70), and Ter119 (TER-119) were applied. All antibodies were purchased from eBiosciences (San Diego, CA, USA), Miltenyi Biotec (Bergisch Gladbach, Germany), or produced in-house (DRFZ, Berlin). A total of 2 × 10^6^ cells were stained in 100 *μ*L for 30 minutes on ice in the dark. Data were acquired on LSRII (BD Biosciences, Heidelberg, Germany) and analyzed by the FlowJo software (Tree Star Inc., Ashland, OR, USA).

### Statistical Analysis

Results were displayed as group means±standard deviation of the mean, or, as quartiles in the box and whisker plots with the median, first, and third quartiles, as well as ranges, indicated. Statistical analysis was performed using the SPSS Software (IBM, Ehningen, Germany) and a *P* value of less than 0.05 was chosen as the significance level for all analyses. The Kolmogorov–Smirnov test was used to establish normal distribution of the data and Levine's test was used to establish normality of variance. Cerebral blood flow and DTI data were compared using two-way repeated measures analysis of variance (ANOVA) with time and region as the two within-subject factors. Water maze data were compared using a mixed design two-way repeated measures ANOVA with surgery (sham versus microcoil) and drug treatment (vehicle versus C21) as the between-subject factors, and time as the within-subject factor. Performance in the probe trial and angiography data were compared using a two-way ANOVA with surgery and drug treatment as the two independent variables.

## Results

### Quantitative Cerebral Blood Flow Imaging Reveals Distinctive Patterns of Perfusion Changes after Microcoil Implantation

**Figure 2:**
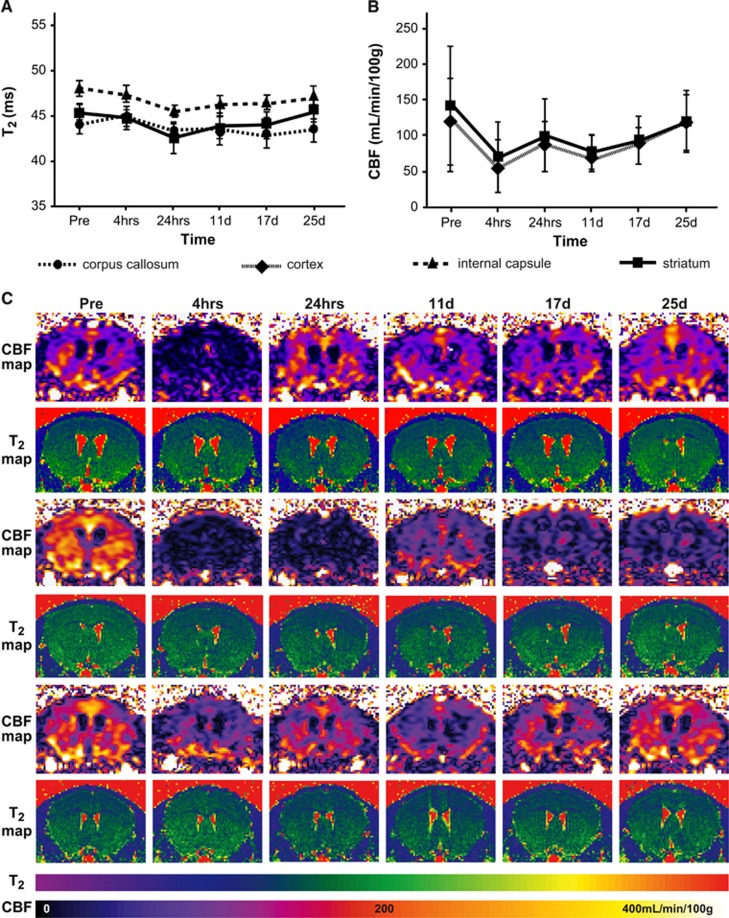
Microcoil implantation resulted in decreased cerebral blood flow (CBF) that partially recovered with time. (**A**) *T*_2_ and (**B**) CBF over time (means±s.d.) in the hypoperfused mouse brain (*n*=8). While *T*_2_ remained relatively stable, CBF decreased immediately after microcoil implantation and subsequently increased over 3 weeks. (**C**) CBF and *T*_2_ maps of three individual animals indicate different patterns of CBF change. Note: the scale bars correspond to CBF and *T*_2_ values.

In the first experiment, we monitored changes in *T*_2_ relaxivity and CBF starting 4 hours after microcoil implantation in a separate group of mice that did not undergo behavioral testing. Large increases in *T*_2_ are associated with vasogenic edema and pathology, but *T*_2_ was relatively stable over time in all three measured VOIs: corpus callosum, internal capsule, and striatum (Figure 2A). Due to the lower resolution of the CBF maps, we were only able to isolate two ROIs: the cortex and the striatum. Nevertheless, CBF decreased immediately after microcoil implantation (by more than half) in both ROIs and subsequently increased to near presurgical levels after approximately 3 weeks (main effect of time F(5,35) 2.753, *P*=0.034, region F(1,7) 11.886, *P*=0.011, and interaction F(5,35) 2.737, *P*=0.034); Figure 2B). Interestingly, three individual patterns of CBF change were identified (Figure 2C). The first was a dramatic decrease immediately after microcoil implantation that either recovered (top panel), or remained low (middle panel). The remaining third of the mice exhibited only a small initial decrease in CBF followed by little fluctuation for the duration of the experiments (bottom panel). Interestingly, one animal showed a delayed decrease in CBF starting around 11 days. Though this animal did not meet the predetermined exclusion criteria, the possibility exists that this response could be an outlier.

### Diffusion Tensor Imaging Reveals White-Matter Degeneration after 3 Weeks of Hypoperfusion

**Figure 3:**
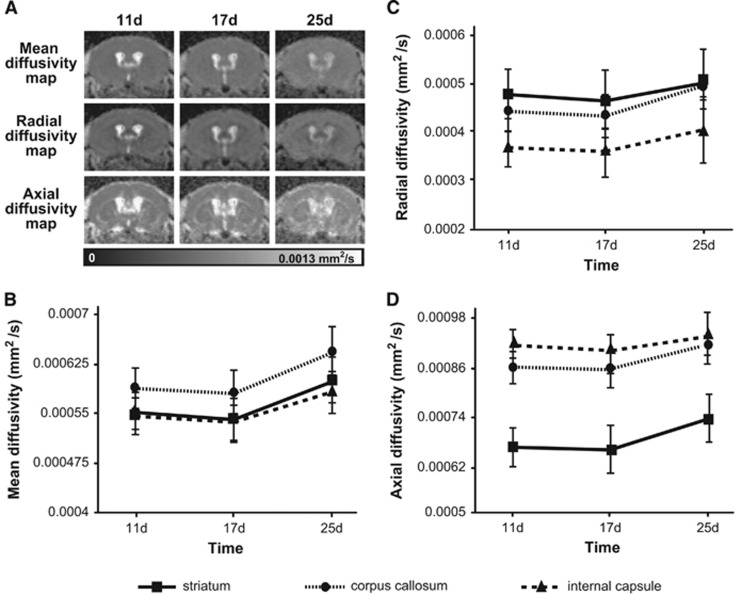
Microcoil implantation increased several diffusion tensor imaging (DTI) parameters, which are indicative of white-matter degeneration. (**A**) Mean diffusivity (MD), axial diffusivity (AD), and radial diffusivity (RD) maps over time in a representative animal. (**B**) MD, (**C**) RD, and (**D**) AD over time (means±s.d.) in the hypoperfused mouse brain (*n*=8). Note: the scale bar in (**A**) corresponds to MD, AD, and RD values.

Diffusion tensor imaging is sensitive to water motion, which is faster parallel (rather than perpendicular) to structures like axons. Directional-dependent water diffusion is called anisotropy, thus, white matter is generally identified by strong fractional anisotropy (FA). Decreases in FA mean there is a directional-dependent decrease in water diffusion, and the most common explanation for this is white-matter damage. While FA has been shown to be a useful parameter, measuring the entire diffusion tensor can provide more detailed characterization of tissue microstructure and pathology.^12^ Therefore, we measured changes in several quantitative DTI parameters in the hypoperfused mouse brain: FA, and mean, axial, and radial diffusivity (MD, AD, and RD, respectively). There were no significant changes in FA over time in any of the three VOIs (internal capsule, corpus callosum, or striatum) out to 25 days after microcoil implantation, though there was a significant main effect of region (F(2,12) 695.55, *P*=0.001) as FA values were higher in the white-matter structures when compared with the striatum (data not shown). We observed significant main effects of region and time, but no interactions, for all other DTI parameters: MD, AD, and RD. In general, structural differences in all three parameters were apparent throughout the measurement period, but parameters values increased in all three VOIs, though to different degrees, between the third and fourth postsurgical week (Figure 3). The increase in MD (the overall mean diffusion, independent of direction) was the most dramatic (Figure 3B; main effect of time (F(2,12) 11.067, *P*=0.002) and region (F(2,12) 81.858, *P*=0.0001)), implying an overall reduction in tissue integrity in all three VOIs. Overall increases in RD (diffusion perpendicular to the main direction) were less profound (Figure 3C; main effect of time (F(2,12) 12.169, *P*=0.001) and region (F(1.1,6.6) 238.9, *P*=0.0001)). However, they occurred to the highest degree in the corpus callosum and internal capsule, which is not surprising because both are white-matter structures and RD increases are correlated with demyelination. Decreases in AD (diffusion parallel to the main direction) have been correlated with axonal degeneration. Interestingly, we observed increases in AD (Figure 3D; main effect of time (F(2,12) 6.592, *P*=0.012) and region (F(2,12) 345.23, *P*=0.0001)) in all three structures and these effects were most profound in the striatum.

### Compound 21 Treatment Has Opposing Effects on Spatial Reference Memory in Sham and Microcoil Mice

**Figure 4:**
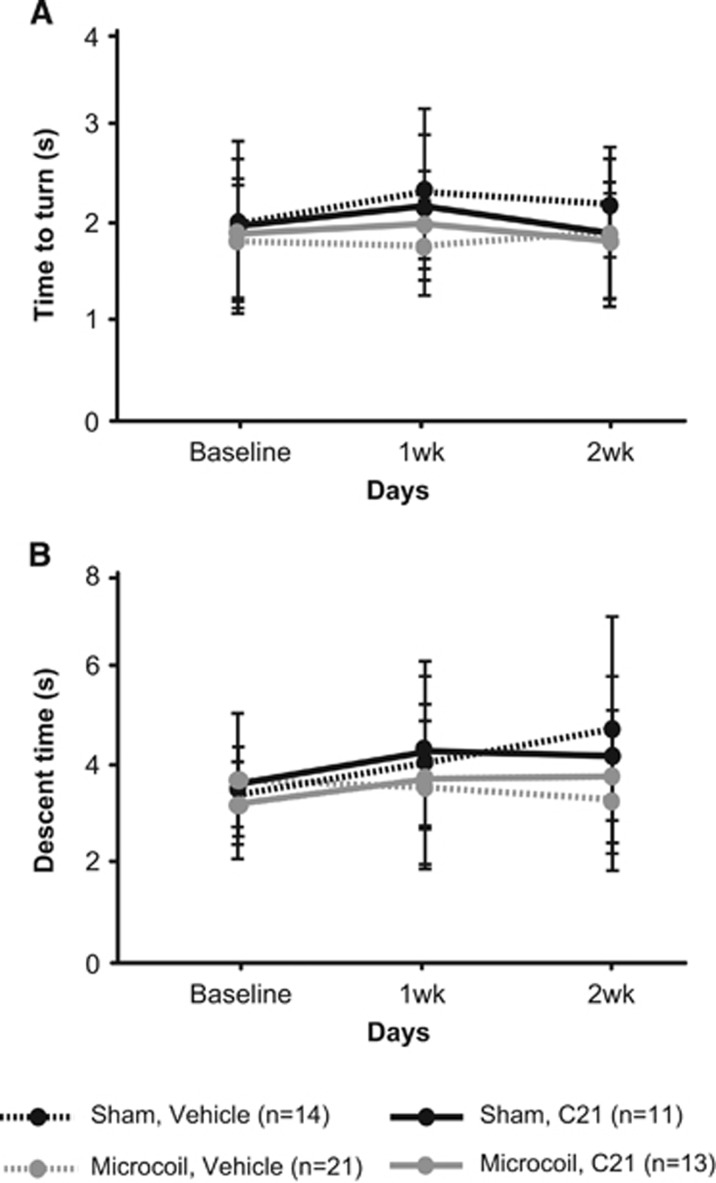
Hypoperfusion, irrespective of Compound 21 (C21) treatment, did not induce sensorimotor deficits. (**A**) Time to turn and face downwards (means±s.d.) and (**B**) time to descend the pole into the home cage in all four groups.

**Figure 5:**
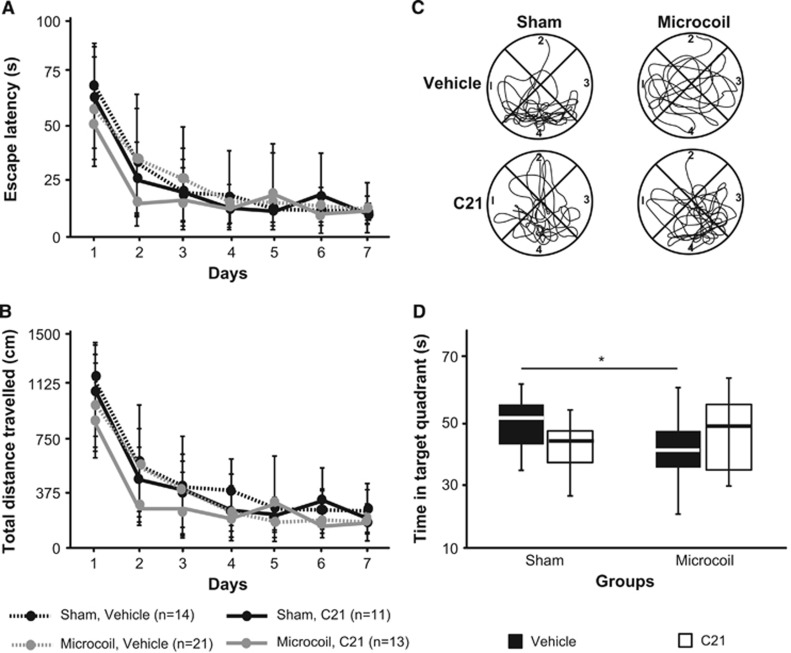
The effects of hypoperfusion and Compound 21 (C21) on spatial learning and reference memory. (**A**) Escape latency and (**B**) total distance travelled by each group during the place task in the Morris water maze (means±s.d.). (**C**) Representative swim paths from an animal in each group and (**D**) total time spent in the target quadrant (#4) during the probe trial. Note: The * corresponds to *post-hoc* Bonferonni corrected multiple comparisons.

We aimed to examine sensorimotor and cognitive function in hypoperfused mice with daily intraperitoneal administration of either C21 or vehicle, starting at the time of surgery. No sensorimotor impairments were observed in the pole test after microcoil implantation, irrespective of treatment or surgery. All mice were equally able to turn around on the pole (Figure 4A) and descend into the home cage (Figure 4B). None of the groups exhibited impairments in spatial learning in a water maze place task (Figures 5A and 5B). All groups acquired the paradigm over 7 days and thus exhibited decreased escape latencies (main effect of time F(3.809,209.481) 88.935, *P*=0.0001) and travelled less distance to find the submerged platform (main effect of time F(3.941,216.771) 103.498, *P*=0.0001). There were no differences in swim speed among the different groups (data not shown), indicating again comparable sensorimotor abilities. When the platform was removed during the probe trial on the final test day, there were no significant main effects (for surgery or treatment). However, there was a significant interaction between surgery and treatment (F(1,55) 5.336, *P*=0.025; two-way ANOVA). Less time in the target quadrant is used as an indicator of impaired spatial reference memory. Interestingly, C21 had a positive effect on microcoil mice (increased the amount of time spent in the target quadrant), but had a negative effect on sham mice (decreased the amount of time spent in the target quadrant) (Figures 5C and 5D).

### Hypoperfusion Induced Arterial Remodelling of the Anterior Region of the Circle of Willis

**Figure 6:**
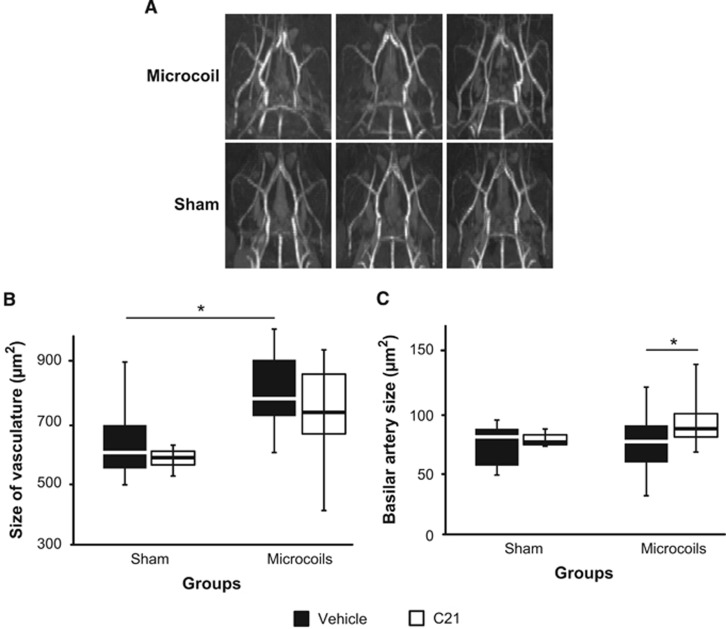
The effects of hypoperfusion and Compound 21 (C21) on vascular remodelling. (**A**) Representative maximum intensity projections of the Circle of Willis in sham and microcoil animals. (**B**) Overall size of the Circle of Willis vasculature and (**C**) basilar artery (Sham, Vehicle (*n*=14); Sham, C21 (*n*=3); Microcoil, Vehicle (*n*=20); Microcoil, C21 (*n*=11)). Note: The * corresponds to *post hoc* Bonferonni corrected multiple comparisons.

Bilateral common carotid artery occlusion in rats results in vascular remodelling in the major cerebral arteries in response to sheer stress,^13^ particularly the basilar artery.^14^ We used magnetic resonance angiography to monitor changes in the arterial network of hypoperfused mice treated with C21 or vehicle. Microcoil mice exhibited increases in arterial tortuosity and diameter that were most prominent in the anterior portion of the Circle of Willis, rather than the basilar artery (Figure 6A). There was a significant main effect of surgery (F(1,44) 12.005, *P*=0.001) in the overall size of the Circle of Willis vasculature. The largest overall arterial networks were observed in the microcoil vehicle-treated mice when compared with the sham vehicle-treated mice (Figure 6B). The effects of hypoperfusion on the basilar artery were less profound, which is not surprising given the lack of collateralization in mice; there was no significant main effect of surgery. However, the main effect of treatment just reached significance (F(1,44) 4.110, *P*=0.047). *Post hoc* comparisons revealed a treatment difference only in the microcoil animals. Those treated with C21 exhibiting slightly larger basilar arteries than those treated with vehicle (Figure 6C). We also calculated ventricle-to-brain ratio from the *T*_2_-weighted images, which is generally used as an overall indicator of brain atrophy. Ventricle-to-brain ratio was reasonably comparable among the groups despite high variability (data not shown), suggesting no gross brain atrophy in any of the mice.

### Compound 21 Treatment Promoted Lymphocyte Infiltration into the Brain

We used flow cytometry to estimate the proportion of lymphocytes, and their subtypes, in the brain and blood from animals in each group at the conclusion of the experiments. Interestingly, large increases in T and B cells and macrophages were observed in the brains of all C21-treated animals (microcoil and sham) compared with the corresponding vehicle-treated groups (Table 1). This suggests that the microcoil model itself does not produce a systemic inflammatory response, but that treatment with C21 is capable of recruiting leukocytes to the brain. The same was true for CD4+ T cells and B cells in the blood samples (data not shown).

## Discussion

This study identified different patterns of CBF change in a mouse chronic hypoperfusion model, and increases in DTI indices that suggest early white-matter degeneration in the brain. We also showed that the AT2R agonist C21 differentially influenced spatial reference memory in hypoperfused and sham mice. While we cannot confirm the exact mechanism behind this effect, it could, in part, be mediated by increased brain lymphocyte infiltration, which was observed after C21 treatment. Overall, our results provide important outcome measures for future work with this model, and suggest a role of the AT2R in VCI.

Vascular risk factors represent the first step in the pathogenesis of VCI. While we are the first to use MRI to quantitatively report on CBF changes in a mouse model of VCI, several groups have already applied this technique in different strains of Alzheimer's disease mice, which highlights the importance of CBF imaging to study vascular dysfunction in both Alzheimer's disease and VCI.^15, 16^ Cerebral blood flow has been semiquantified in the cortex of hypoperfused mice using Laser Doppler Flowmetry, and an acute 30% decrease was noted that also increased to near presurgical levels within a month.^3^ Our quantitative measurements agree with this, only we observed a greater initial decrease (approximately 50%) that was also present in subcortical structures. Both studies reported a high degree of variability in the CBF measurements. However, this is not necessarily surprising for Laser Doppler, or for FAIR EPI-based measurements that are extremely difficult to perform in the small mouse brain. With this technique in the mouse, there is a very high level of noise, the standard deviation of the noise is approximately 25%. This makes it difficult to accurately measure the small difference in *T*_1_ signal between the selective and nonselective experiments. Despite making effort to maintain a constant level of anesthetic, there may have also been slight differences in physiologic parameters that contributed to interanimal variability. One of the most important findings of the present work is that we were able to identify different patterns of CBF change among hypoperfused animals, which highlights the utility of CBF imaging as an outcome measure in this model. It is possible that some of the animals that exhibited CBF recovery may not have had sufficient maintenance of the induced stenosis. Indeed, we can not exclude this possibility as the high amount of fibrous deposits on the coils and arteries prevented extraction of the carotids to examine them histologically. We also performed DTI in the same group of hypoperfused mice to obtain an additional outcome measure. White-matter hyperintensities in *T*_2_-weighted or FLAIR images are a useful hallmark of certain VCI subtypes. Furthermore, DTI is a predictor of cognitive decline in patients with leukoaraiosis,^17^ and is a promising technique to identify presymptomatic microstructural changes in white matter.^18^ We measured significant increases in MD, RD, and AD in several brain structures. It is well accepted that MD and RD increases represent a loss of tissue integrity and demyelination, respectively;^19^ the present study confirms this. While MD increases were present in all measured structures, implying an overall loss of tissue integrity, the greatest increases in RD occurred in the white-matter structures (internal capsule and corpus callosum) when compared with the striatum (a mixture of white and gray matter). Other studies have reported that decreases in AD were correlated with axonal injury.^15, 20^ We observed instead an increase in AD, particularly in the corpus callosum and striatum. This may be due to the fact that AD can be influenced by axonal swelling and/or transport disruption that mask axonal injury.^20^ However, a DTI study of patients with small vessel disease noted increases in AD, which were interpreted as arteriopathy unique to lacunar infarction and leukoaraiosis.^21^ The increase in AD may also imply that there is little axonal injury in this model. This is in agreement with the only other DTI study in hypoperfused mice that observed evidence of demyelination (instead of axonal injury) that was correlated with a subtle decrease in FA in the corpus callosum and the internal capsule.^22^ We did not observe a decrease in FA. This discrepancy may be the result of differences in sequence parameters; the other group did not use isotropic voxels, which makes it difficult to accurately measure diffusivity indices. However, the lack of decrease in FA may not be entirely surprising as FA is a gross measure of anisotropy. Diffusivity indices, particularly MD, have been shown to be better predictors of small vessel disease.^21^ We attempted to correlate the degree of CBF deficit at 4 hours with the AD diffusivity values at 25 days, as we hoped that the initial CBF decrease would be a useful predictor of final outcome. However, no correlation was observed. This may be due to the relatively small sample size, or the fact that there was a very small dynamic range of observed DTI values. This remains a limitation of the present study and future work is required to improve the predictive ability of the model.

While spatial working memory impairments have been reported in microcoil mice in the radial arm maze, spatial learning in the water maze has remained intact.^4, 5^ Our results are in agreement, except that we found spatial reference memory impairments in the water maze during the probe trial. It should be noted that this was not consistently observed, which again suggests this model is more variable than previously reported. The most interesting finding of the present study was the significant interaction between surgery and treatment during the probe trial. Compound 21 both improved and impaired spatial reference memory in microcoil and sham animals, respectively, suggesting pleotropic effects on cognitive function. Another study reported C21 enhanced cognitive function in normal mice because spatial learning, but not reference memory, was improved in the water maze.^9^ In the same study, spatial learning improvements were detected on day 5 of water maze training in C21-treated Alzheimer's mice. The authors speculated these effects were mediated by enhancement of hippocampal excitatory postsynaptic potentials. Similar findings were reported when C21 treatment was combined with memantine (Alzheimer's treatment) in diabetic mice.^23^ While we did not examine neurotrophic processes in the hippocampus, we did observe a subtle increase in the size of the basilar artery in C21-treated microcoil mice. The blood supply to the hippocampus is provided by the posterior cerebral artery, which branches off the superior cerebellar from the basilar artery. Therefore, it is possible that the larger basilar arteries observed in hypoperfused C21-treated animals may have resulted in a slightly improved blood supply to the vulnerable hippocampus, thus assisting with the preservation of spatial reference memory in this group when compared with the vehicle-treated animals. While C21 does not influence blood pressure, it has been shown to induce vasodilation in rat and mouse arteries via an AT2R receptor-independent mechanism.^24^ We also observed an increase in lymphocyte infiltration into the brains of C21-treated mice. It is possible that these infiltrating cells may have partially influenced the differential effects we observed in spatial reference memory. Indeed, a recent study showed that cardiovascular injury, in addition to inducing potentially cytotoxic CD8+ T cells, also promoted the release of subpopulations of T cells that expressed AT2R.^25^ These AT2R+ T cells were capable of producing IL-10 and were protective in another cohort of animals with myocardial infarction. Interestingly, another study reported that low doses of C21 do not influence leukocyte migration to the stroke brain, though administration did result in neuroprotection, likely due to the upregulation of inducible nitric oxide synthase that would have reduced production of the damaging free radical nitric oxide.^26^

In conclusion, we have successfully applied neuroimaging to identify several new outcome measures in a mouse model of chronic hypoperfusion, these outcome measures are applicable in humans, and may serve to facilitate translation of knowledge between bench and bedside. We have also shown beneficial effects of C21 on spatial reference memory only in hypoperfused mice, though the exact mechanism still requires investigation, this suggests that the RAS has a role in VCI.

## Figures and Tables

**Table 1:** Increased lymphocyte infiltration into the brains of C21-treated animals

	*T cells (CD3+)*		*B cells (CD3-)*	*Macrophages (CD3-)*
	*CD8+*	*CD4+*	*CD19+*	*Mac-1+*
Sham, Vehicle	13.7±1.6	1.5±0.7	21.6±6.8	5.4±1.6
Sham, C21	29.7±2.8	48.0±3.9	40.4±4.8	20.5±2.1
Microcoil, Vehicle	16.8±4.1	7.5±11.0	11.8±5.3	7.2±2.0
Microcoil, C21	28.7±2.2	50.7±2.4	42.1±10.3	20.1±4.9

C21, Compound 21. Expressed as a percentage of the lymphocyte parental cell population (*n*=4 per group).
